# Optimization, Characterization and Pharmacological Validation of the Endotoxin-Induced Acute Pneumonitis Mouse Model

**DOI:** 10.3390/biomedicines13061498

**Published:** 2025-06-18

**Authors:** Emese Ritter, Kitti Hohl, László Kereskai, Ágnes Kemény, Dóra Hargitai, Veronika Szombati, Anikó Perkecz, Eszter Pakai, Andras Garami, Ákos Zsembery, Zsuzsanna Helyes, Kata Csekő

**Affiliations:** 1 Department of Pharmacology and Pharmacotherapy, Medical School, University of Pécs, Szigeti út 12, H-7624 Pécs, Hungary; rittermesi@gmail.com (E.R.); veronika.szombati@aok.pte.hu (V.S.); perkecz@gmail.com (A.P.);; 2 Department of Pathology, Medical School, University of Pécs, Szigeti út 12, H-7624 Pécs, Hungary; 3 Department of Pathology, Forensic and Insurance Medicine, Faculty of Medicine, Semmelweis University, Üllői út 93, H-1091 Budapest, Hungary; hargitai.dora@semmelweis.hu; 4 Department of Thermophysiology, Institute for Translational Medicine, Medical School, University of Pécs, Szigeti út 12, H-7624 Pécs, Hungaryandras.garami@aok.pte.hu (A.G.); 5 Department of Oral Biology, Faculty of Dentistry, Semmelweis University, Nagyvárad tér 4, H-1089 Budapest, Hungary; zsembery.akos@semmelweis.hu; 6 National Laboratory for Drug Research and Development, Magyar Tudósok krt. 2, H-1117 Budapest, Hungary; 7 HUN-REN-PTE Chronic Pain Research Group, H-7624 Pécs, Hungary; 8 PharmInVivo Hungary Ltd., Szondy György u 10, H-7629 Pécs, Hungary

**Keywords:** acute lung injury, LPS, dexamethasone, C57BL/6J, NMRI, airway function, interstitial pneumonitis

## Abstract

**Background/Objectives**: In preclinical research of airway inflammation, the endotoxin (lipopolysaccharide: LPS)–induced acute interstitial pneumonitis is the most commonly used mechanism model. However, studies apply different LPS serotypes, doses, administration routes, and reference compounds, making result interpretation challenging and drawing conclusions difficult. Therefore, here we aimed to optimize, characterize, and validate this model with dexamethasone in mice. **Methods**: Pneumonitis was induced by intratracheal LPS (0.25, 1, 2.5, 5 mg/kg; *E. coli* O111:B4) in C57BL/6J and NMRI mice; controls received phosphate-buffered saline (PBS). Dexamethasone (5 mg/kg i.p.) was used as a positive control. Respiratory functions were measured by restrained plethysmography 24 h after induction, and core body temperature was monitored. Lungs were excised and weighed, and then myeloperoxidase (MPO) activity and histopathological analysis were performed to assess pulmonary inflammation. **Results**: LPS-induced significant body weight loss, perivascular pulmonary edema, MPO activity increase, neutrophil infiltration, and respiratory function impairment in a dose-independent manner. However, LPS-induced hypothermia dynamics and duration were dose-dependent. The inhibitory effects of the reference compound dexamethasone were only detectable in the case of the 0.25 mg/kg LPS dose on most inflammatory parameters. These results did not differ substantially between C57BL/6J and NMRI mouse strains. **Conclusions**: Very low doses of LPS induce characteristic functional and morphological inflammatory alterations in the lung, which do not worsen in response to even 20 times higher doses. Since the effect of pharmacological interventions is likely to be detectable in the case of the 0.25 mg/kg LPS dose, we suggest this protocol for testing novel anti-inflammatory agents.

## 1. Introduction

Acute lung injury (ALI) and its more severe form, acute respiratory distress syndrome (ARDS), are characterized by diffuse structural and functional impairment with an extremely high 40% mortality rate among critically ill patients [1,2]. The progression of ALI/ARDS is defined by an acute inflammation leading to endothelial-epithelial barrier damage with an increased microvascular permeability, excessive inflammatory cell infiltration, and edema formation in the alveoli [3]. It is mainly initiated by various direct damaging factors on the lung, including aspiration, contusion, mechanical damage, pneumonia, and/or indirect stimuli, such as systemic inflammatory conditions, sepsis, pancreatitis, and severe burns.

Due to the COVID-19 pandemic and the high ARDS complications of SARS-CoV-2 infected patients paralyzing the global health care system in 2020, ALI/ARDS has become the focus of attention, as shown by the number of publications having skyrocketed in the field [4,5]. The gold standard therapy is glucocorticoids; however, its effect is not consistent. Many patients do not respond to this treatment, which might be partially attributable to the improper time of administration and dose [6]. Therefore, in a fierce race against time, many drug repositioning clinical studies have been launched to develop the most efficient guidelines to improve the overall survival [7]. Most studies have been critically received, many initial therapeutic recommendations have been revoked, and the treatment of glucocorticoid-resistant ARDS is still unresolved [8,9].

It is, therefore, especially important to further elaborate the inflammatory cascades and identify novel drug candidates, for which optimized, characterized, and validated preclinical models are inevitable. Various animal models are available to investigate the underlying mechanisms of ALI [10]. The endotoxin (lipopolysaccharide: LPS)-induced acute interstitial pneumonitis model is a well-defined, reproducible, and standard acute airway inflammation model in preclinical research [11]. LPS in the outer membrane of Gram-negative bacteria induces a macrophage-driven, neutrophil-dominant inflammatory response through pro-inflammatory cytokine production via the toll-like receptor 4 (TLR4) [10,12]. As such, the model is suitable for preclinical testing of novel drug candidate molecules if optimized and properly validated. Although this model is commonly used to test novel drug candidates, its variability is rather great, since different studies often apply different LPS serotypes, doses (0.25–15 mg/kg), routes of administration (intratracheal, intranasal, and inhalation), species/strains and most often the lack of a positive control reference compound limiting the interpretation and comparison of the results (Table 1).

We, therefore, aimed to optimize and validate the model in C57BL/6J and NMRI mice based on the most common protocol variables. 

## 2. Materials and Methods

### 2.1. Animals

Experiments were performed on 8–10-week-old female C57BL/6J (20 ± 3 g) and Crl:NMRI(Han) (NMRI) (30 ± 2 g) mice. Both strains were purchased originally from the Charles River Laboratories, Calco, Italy, via Animalab Ltd., Vác, Hungary, bred and kept in the minimal disease (MD) Laboratory Animal House of the Department of Pharmacology and Pharmacotherapy, located in the Preclinical Research Center, University of Pécs. Animals kept in the MD facility are non-pathogen free; the microbiological status of mice is monitored regularly by GVG Diagnostics GmbH, Leipzig, Germany, based on FELASA recommendations. Animals are bred for 10 generations and renewed by introducing new stock provided by the original vendor. Altogether 109 C57BL/6J (F5 generation) and 44 NMRI (F4 generation) mice were used in the study, given standard laboratory chow (SAFE^®^ A03 pellets, SAFE^®^, Augy, France) and tap water ad libitum. Animals were housed in autoclaved open-type TII cages (367 × 140 × 207 mm) (Acéllabor Ltd., Vecsés, Hungary) on spruce wood fibrillated fibers (SAFE^®^ 3-4 S, SAFE^®^, Augy, France) enriched by GLP fun tunnels (Innovo Ltd., Isaszeg, Hungary). LPS-treated animals were housed in a separate room of the Animal House. Standardized conditions were provided at constant temperature (20–22 °C), 40–55% humidity, and a 12-h light-dark cycle switched on at 6 a.m. and off at 6 p.m. Animal welfare was assessed by the mouse grimace scale throughout the study.

### 2.2. Materials

Bacterial LPS was purchased from Merck (*Escherichia coli* endotoxin O111:B4 (catalogue no. L2630); Merck KGaA, Darmstadt, Germany). Dexamethasone was obtained from Teva (Dexa-ratiopharm 4 mg/mL; Teva Pharmaceuticals Ltd., Debrecen, Hungary). The myeloperoxidase (MPO) reagent was purchased from Merck (MPO from human polymorphonuclear leukocytes, CAS number: 9003-99-0; Merck KGaA, Darmstadt, Germany).

### 2.3. Experimental Protocol of Endotoxin-Induced Acute Interstitial Pneumonitis Model

**Figure 1:**
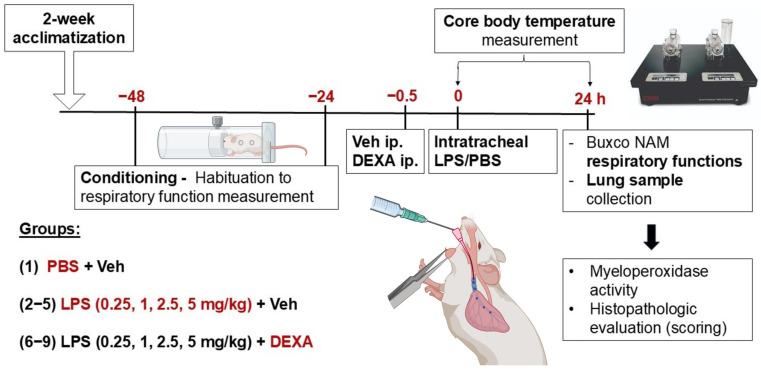
Experimental design of LPS-induced acute pneumonitis model. (PBS: phosphate-buffered saline, LPS: lipopolysaccharide, Veh: Vehicle (physiological saline solution), DEXA: dexamethasone). Created in BioRender. Bencze, N. (2025) https://BioRender.com/0odkhaf (accessed on 3 April 2025).

Acute airway inflammation was induced by intratracheal administration of 4 different doses (0.25 mg/kg, 1 mg/kg, 2.5 mg/kg, and 5 mg/kg) of LPS (5, 20, 50, 100 μg dissolved in 60 μL phosphate-buffered saline (PBS)) in C57BL/6J mice. Animals were randomized into nine treatment groups (Figure 1): (1) negative control group (PBS); (2–5) LPS (0.25 mg/kg, 1 mg/kg, 2.5 mg/kg, and 5 mg/kg) + i.p. saline (vehicle: Veh); (6–9) positive control group receiving LPS (0.25 mg/kg, 1 mg/kg, 2.5 mg/kg, and 5 mg/kg) + the reference compound dexamethasone (DEXA, 5 mg/kg i.p.) 30 min prior to induction, as elaborated in Supplementary Table S5. Respiratory function measurements were performed 24 h after LPS treatment, and then animals were anesthetized with an intraperitoneal injection of ketamine and xylazine (100 mg/kg and 5 mg/kg, respectively). After the lungs were harvested and measured, the right lung was frozen in liquid nitrogen and stored for further evaluation of MPO activity, and the left lobe was formalin-fixed for histopathological assessment. Based on the previously described experiment, the most optimal LPS dose (0.25 mg/kg) was chosen to validate the model in the NMRI strain, as elaborated in Supplementary Table S5.

### 2.4. Induction of Acute Pneumonitis by Intratracheal Administration of LPS

Acute lung injury was induced by intratracheal administration of 0.25 mg/kg, 1 mg/kg, 2.5 mg/kg, and 5 mg/kg LPS in animals anesthetized by injection of 100 mg/kg ketamine and 5 mg/kg xylazine intraperitoneally. LPS or PBS solutions were injected through a BTPE-10 cannula (0.28 × 0.60 mm, Instech Laboratories, Inc., Leipzig-Markkleeberg, Germany) into the trachea over 2–3 s. Animals were kept on a heating pad (model: V500DVstat; PECO Services Ltd., Brough, UK) to prevent hypothermia during anesthesia, and to prevent corneal damage, hydrophilic eye ointment mixed in the Univ Pharmacy (University of Pécs Clinical Center, Pécs, Hungary) was applied. Vital signs were monitored until awakening.

### 2.5. Pulmonary Function Measurement with Buxco Non-Invasive Airway Mechanics Plethysmometry

Respiratory functions were measured in awake animals using Buxco FinePointe Non-Invasive Airway Mechanics (NAM) restrained full body plethysmography (DSI Harvard Bioscience Inc., St. Paul, MN, USA) 24 h after LPS/PBS administration. Respiratory function parameters (breathing frequency, tidal volume, minute ventilation, peak expiratory flow rate) were recorded every 2 s for 5 min. Baseline measurement was preceded by a 15-min acclimatization period to ensure the animals adapted to immobilization.

### 2.6. Investigation of Core Body Temperature

An additional set of experiments with LPS-treated animals was conducted to record abdominal temperature (T_ab_, a measure of deep body temperature) changes in the model, as elaborated in Supplementary Table S5. Mice were implanted with a miniature telemetry transmitter (G2 E-Mitter series; Mini Mitter, Bend, OR, USA) 7 days before LPS induction. Animals were anesthetized with i.p. administration of a ketamine-xylazine and received antibiotic protection intramuscularly (gentamycin, 6 mg/kg). A hydrophilic eye ointment mixed in the Univ Pharmacy (University of Pécs Clinical Center, Pécs, Hungary) was applied to prevent corneal damage during anesthesia. After shaving the abdomen and sterilizing the surgical area with 7.5% povidone-iodine solution, the device was inserted into the peritoneal cavity via a small midline incision (15 mm) and fixed to the left side of the abdominal wall with suture (type 3-0 non-absorbable sterile silk suture, Kruuse, Denmark). The surgical wound was sutured in layers, and the procedure lasted for 15–20 min. To prevent intra- and postoperative hypothermia, mice were kept on a heating pad set to 38 °C (model: V500DVstat; PECO Services Ltd., Brough, UK) and then were allowed to recover from anesthesia in a temperature-controlled chamber (model BJPX-B400II; Biobase; Jinan, China) set to an ambient temperature of 28.0 °C. Experiments were conducted in the telemetric thermometry setup. Telemetry receivers (model ER-4000; Mini Mitter) were positioned in the room, and the home cage of each mouse was placed on top of a receiver.

### 2.7. Histopathological Assessment of the Lungs

Semiquantitative morphological evaluation was performed on hematoxylin-eosin (HE)-stained 5 µm sections from three distinct depths and areas of lung samples. Scores were determined by two independent pathologists in a blinded manner and averaged using the following criteria: perivascular edema, peribronchial and perivascular neutrophilic granulocyte accumulation, and eosinophilic macrophage infiltration (0: none, 1: mild, 2: moderate, and 3: severe). The composite score was calculated as the sum of the mean scores (0–9) as described previously [41].

### 2.8. Determination of MPO Activity from Lung Homogenates

MPO activity, indicative of the accumulation of activated neutrophil granulocytes and macrophages and, consequently, the extent of inflammation, was assessed by spectrophotometric measurement of lung homogenate samples as described previously [42].

### 2.9. Statistical Analysis

Statistical evaluation of all data, with the exception of core body temperature, was performed by effect size analysis. Values of all measurements are expressed as means ± SEM. All data with a high effect size, indicated by Hedges’ g value exceeding 0.8, were considered significant and marked on the figures. Moreover, all data was further evaluated by GraphPad Prism v8 (GraphPad Software, San Diego, CA, USA) statistical analysis software using either one-way ANOVA followed by Dunnett’s multiple comparisons test (lung weight, respiratory function parameters, and MPO activity), two-way ANOVA followed by Tukey’s post hoc test (body weight change and T_ab_) or Kruskal–Wallis followed by Dunn’s post hoc test (histopathological semiquantitative scores), as well as linear regression analysis for the evaluation of dose-dependence (with significance *p* < 0.05). Data points in the respiratory function and MPO activity measurements were only excluded due to technical errors, as elaborated in Supplementary Table S6.

## 3. Results

### 3.1. Dexamethasone Can Diminish LPS-Induced Body Weight Loss and Increased Lung Index Depending on the LPS Dose

**Figure 2:**
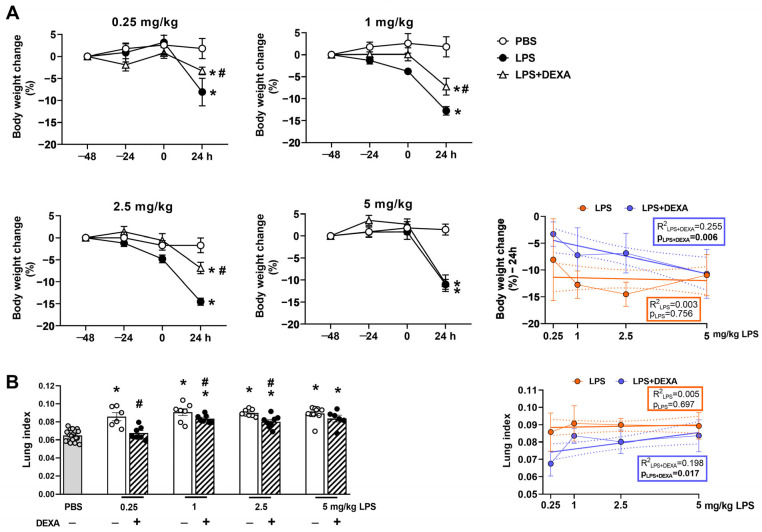
Alterations in LPS-induced (**A**) body weight change and (**B**) lung index (total lung weight/10 g body weight) in response to dexamethasone. DEXA treatment counteracted 0.25, 1, and 2.5 mg/kg LPS-induced body weight loss, and increased lung index but did not influence high-dose LPS (5 mg/kg)-evoked alterations. The line graph represents a simple linear regression analysis of the respective parameters. n = 6–18/group (Effect size analysis, * Hedges’g > 0.8 vs. PBS; # Hedges’g > 0.8 corresponding LPS group, orange: LPS treatment, blue: LPS + DEXA treatment, thin line: means ± SEM, bold line: best-fit line of linear regression, dotted line: 95% confidence intervals).

Endotoxin induced a significant body weight loss and increase in lung index (total lung weight/10 g body weight) attributed to pulmonary edema in all LPS-treated groups at the 24-h endpoint, which based on our exploratory experiments was determined to be the maximum of the inflammation and related functional alterations declining by 48 h (Supplementary Figure S1, Supplementary Table S1). These parameters were counteracted by dexamethasone dose-dependently (Figure 2, Supplementary Table S2). Approximately 10–15% decrease in body weight was observed in response to LPS administration, which was attenuated by the reference compound in the groups treated with 0.25, 1, and 2.5 mg/kg LPS but had no effect on the 5 mg/kg LPS-treated group (Figure 2A). Dexamethasone prevented lung weight increase only in the 0.25 mg/kg LPS-treated group and attenuated it in the 1 and 2.5 mg/kg LPS-treated groups, whereas it had no effect on the highest LPS dose (Figure 2B).

### 3.2. Dexamethasone Counteracts Endotoxin-Induced Respiratory Function Impairment Depending on the LPS Dose

**Figure 3:**
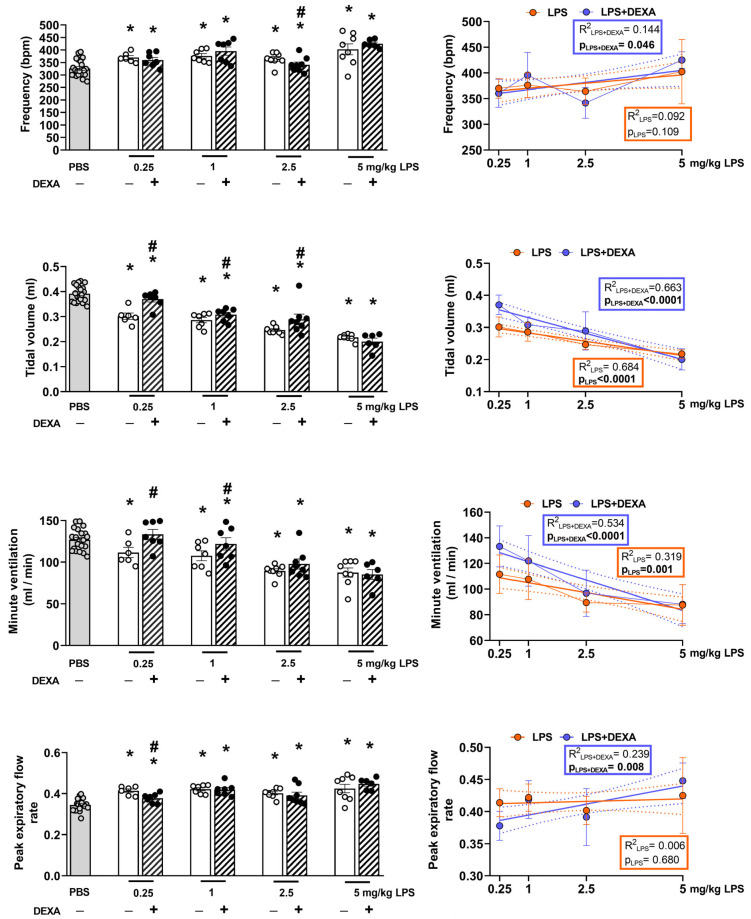
LPS-induced alterations in respiratory functions (frequency, tidal volume, minute ventilation, and peak expiratory flow rate) measured by non-invasive restraint plethysmograph in response to dexamethasone. DEXA treatment counteracted 2.5 mg/kg LPS-induced frequency increase, 0.25, 1, and 2.5 mg/kg LPS-induced tidal volume, as well as 0.25 and 1 mg/kg LPS-evoked minute ventilation decrease, while it only improved peak expiratory flow rate in the lowest, 0.25 mg/kg LPS-treated mice. The line graph represents a simple linear regression analysis of the respective parameters. n = 6–18/group (Effect size analysis, * Hedges’g > 0.8 vs. PBS; # Hedges’g > 0.8 corresponding LPS group, orange: LPS treatment, blue: LPS + DEXA treatment, thin line: means ± SEM, bold line: best-fit line of linear regression, dotted line: 95% confidence interval.).

Respiratory functions, such as breathing frequency and peak expiratory flow rate, increased, whereas tidal volume and minute ventilation decreased to the same extent in response to all applied LPS doses. Dexamethasone’s effect was more pronounced in the lowest LPS-treated group. The reference compound effectively prevented the minute ventilation decrease induced by 0.25 mg/kg LPS and was able to diminish this parameter change in the 1 mg/kg treated group as well. LPS-induced frequency increase was counteracted by dexamethasone only at the 2.5 mg/kg LPS dose, whereas it effectively attenuated tidal volume changes in the groups treated with the lowest (0.25 mg/kg) and intermediate (1 and 2.5 mg/kg) LPS doses. Additionally, the LPS-induced increase in peak expiratory flow rate was counteracted by dexamethasone solely in the group treated with the 0.25 mg/kg dose. None of the respiratory parameters were effectively influenced by dexamethasone in the group treated with high-dose (5 mg/kg) LPS (Figure 3, Supplementary Table S2).

### 3.3. LPS Induces Hypothermia Dose-Dependently

**Figure 4:**
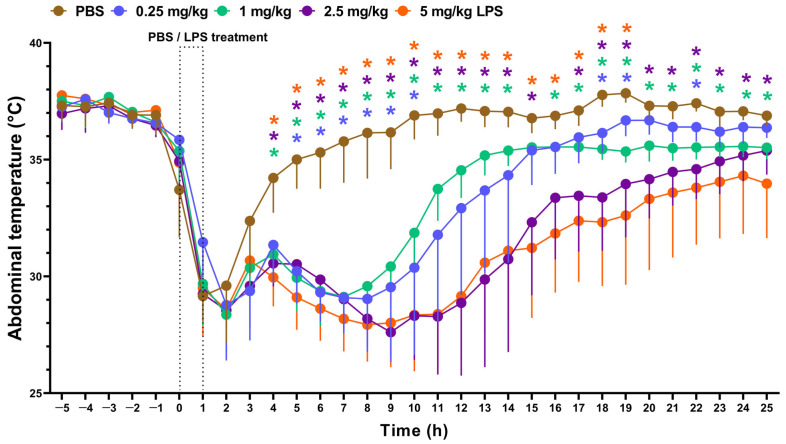
Abdominal temperature changes over the course of 24 h following 0.25, 1, 2.5, 5 mg/kg LPS treatment. n = 6–7/group (2-way ANOVA, Tukey post hoc test, * *p* < 0.05 vs. PBS).

The anesthetic ketamine-xylazine greatly reduced the T_ab_ in all groups. In control animals, the temperature returned to baseline a few hours after administration. However, in the LPS-treated groups, after a transient rise, the T_ab_ decreased and began to converge with the control group in a dose-dependent manner. By 11 h post-induction, the T_ab_ of the low-dose LPS group (0.25 mg/kg) was not significantly different from the control group and recovered approximately at 17 h. (Figure 4, Supplementary Table S3). Although T_ab_ also started to increase in the intermediate LPS (1, 2.5 mg/kg)-treated animals, it still remained significantly lower compared to the PBS-treated control group by the end of the experiment, with the lowest mean values recorded at the maximal LPS dose (5 mg/kg).

### 3.4. LPS-Induced Histopathological Alterations Are Not Dose-Dependent

**Figure 5:**
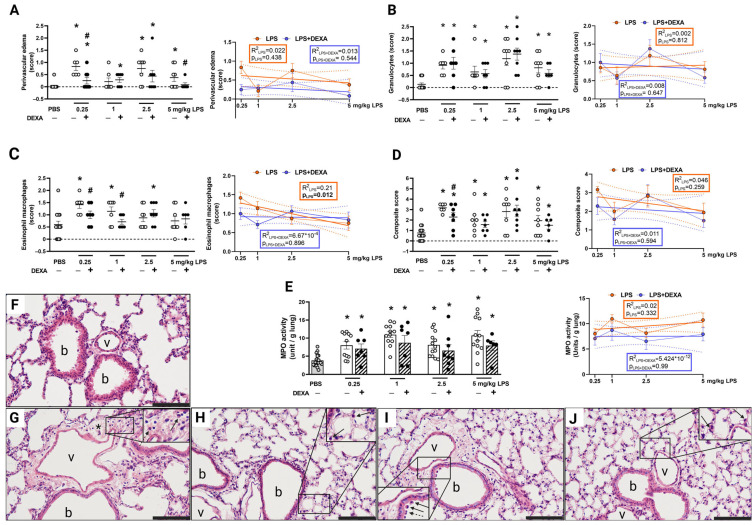
Semiquantitative histopathological evaluation of (**A**) perivascular edema, (**B**) neutrophil granulocyte, (**C**) eosinophil macrophage, (**D**) composite score of lung sections, and alterations in (**E**) MPO activity in response to LPS and dexamethasone. DEXA treatment counteracted 0.25 mg/kg and 5 mg/kg LPS-evoked perivascular edema formation, 1 mg/kg LPS-induced eosinophil macrophage infiltration, while it did not affect either granulocyte infiltration or MPO activity in either LPS dose. The composite score derived from all investigated histological parameters was significantly improved by DEXA only in the lowest applied LPS dose (0.25 mg/kg). The line graphs represent simple linear regression analysis of the respective parameters. n = 6–20/group (Effect size analysis, * Hedges’g > 0.8 vs. PBS; # Hedges’g > 0.8 corresponding LPS group; orange: LPS treatment, blue: LPS + DEXA treatment, thin line: means ± SEM, bold line: best-fit line of linear regression, dotted line: 95% confidence intervals). Representative histopathological pictures of (**F**) control PBS-, (**G**) 0.25 mg/kg LPS-, (**H**) 0.25 mg/kg LPS + DEXA-, (**I**) 5 mg/kg LPS-, (**J**) 5 mg/kg LPS + DEXA-treated lung tissue. (hematoxylin-eosin (HE) staining; 20× magnification; b: bronchiolus, v: vessel, *: edema, arrow: eosinophil macrophage, dashed arrow: neutrophil granulocyte).

Histopathological inflammatory parameters, such as neutrophil and macrophage infiltration, as well as edema formation, were elevated 24 h after LPS administration based on the semiquantitative scores (Figure 5A–D,F–J, Supplementary Table S2). Except for the eosinophil macrophage infiltration, these parameters showed no dose-dependence. Dexamethasone only counteracted the 0.25 and 5 mg/kg LPS-induced edema formation, as well as the 0.25 and 1 mg/kg LPS-evoked eosinophil macrophage infiltration. The composite inflammatory score, which is derived from the sum of the means of the selected histological parameters, was elevated in all LPS-treated groups; however, dexamethasone could effectively alleviate it only in the lowest 0.25 mg/kg LPS group.

### 3.5. LPS-Induced MPO Activity Was Not Influenced by Dexamethasone

MPO activity, which correlates with the number of activated neutrophils and macrophages, was significantly increased by LPS administration at the 24-h endpoint in all groups (Figure 5E, Supplementary Table S2). Dexamethasone did not diminish this parameter significantly at this time point. The regression analysis showed no dose-dependence by either LPS or dexamethasone treatment.

### 3.6. Dexamethasone Counteracts LPS-Induced Increased Total Lung Weight Along with Composite Score and MPO Activity in NMRI Mice

**Figure 6:**
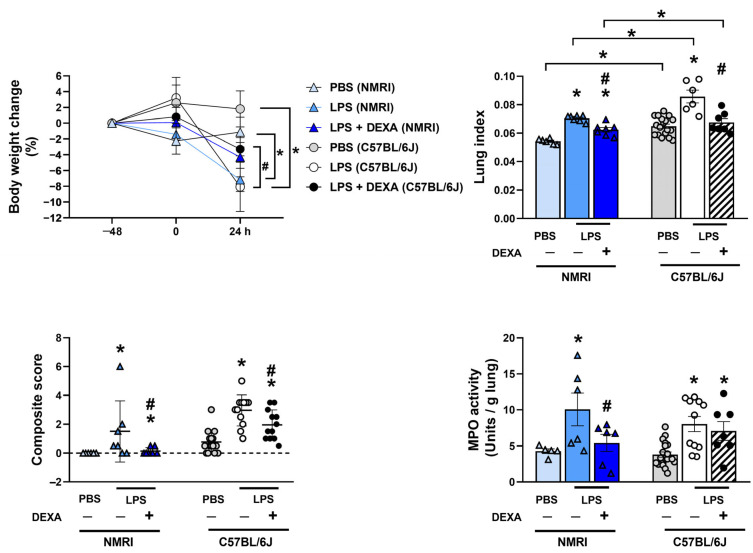
LPS (0.25 mg/kg)-induced alterations in body weight change, total lung weight, composite score, and MPO activity in response to dexamethasone in NMRI compared to C57BL/6J mice. Similar to C57BL/6J mice, DEXA treatment counteracted LPS-induced body weight loss, increased lung index, and increased composite score of histological parameters in NMRI mice; however, it decreased LPS-evoked MPO activity only in the NMRI strain. n = 5–18/group (Effect size analysis, * Hedges’g > 0.8 vs. PBS; # Hedges’g > 0.8 vs. corresponding LPS group).

Based on the previous results with the C57BL/6J mice, our aim was also to validate the 0.25 mg/kg LPS dose in NMRI mice as well. Similar to the results observed in C57BL/6J mice, 0.25 mg/kg LPS induced body weight loss, increased lung weight correlating with pulmonary edema formation, inflammatory histopathological changes represented by the composite score, as well as increased MPO activity in NMRI mice. Dexamethasone diminished LPS-induced lung edema, composite inflammatory score, and decreased MPO activity. However, it did not significantly affect body weight changes, which is due to the difference in the initial body weight of C57BL/6J (20 ± 3 g) and NMRI (30 ± 2 g) mice of the same age (Figure 6, Supplementary Table S4).

### 3.7. Dexamethasone Diminishes LPS-Induced Respiratory Function Alterations in NMRI Mice

**Figure 7:**
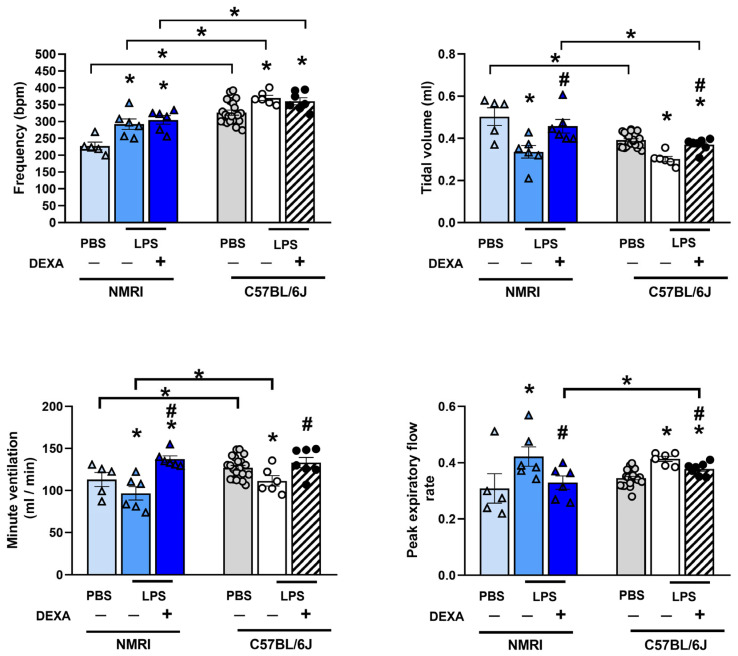
LPS (0.25 mg/kg)-induced alterations in respiratory functions (frequency, tidal volume, minute ventilation, peak expiratory flow rate) in NMRI compared to C57BL/6J mice. Similar to C57BL/6J mice, DEXA treatment did not affect LPS-induced frequency increase but counteracted tidal volume, minute ventilation, and peak expiratory flow rate changes in NMRI mice as well. n = 5–18/group (Effect size analysis, * Hedges’g > 0.8 vs. PBS; # Hedges’g > 0.8 corresponding LPS group).

The baseline frequency, tidal volume, and minute ventilation of PBS-treated control animals showed a strain difference between C57BL/6J and NMRI mice. However, in NMRI mice, 0.25 mg/kg LPS induced a similar increase in breathing frequency and peak expiratory flow rate, along with a decrease in tidal volume and minute ventilation as observed in C57BL/6J mice. Moreover, dexamethasone also prevented the LPS-induced alterations in the latter three parameters in the NMRI strain (Figure 7, Supplementary Table S4).

## 4. Discussion

The main message of the present work is that characterized and pharmacologically validated optimized lung inflammation models are needed for translational research, identifying novel drug targets, and testing compounds. We demonstrated that very low LPS doses induce similar functional and morphological inflammatory pulmonary alterations to 20 times higher doses, but only the effect of 0.25 mg/kg LPS is the most sensitive to the gold standard glucocorticoid treatment. Therefore, this protocol is suggested to be used for controlled preclinical studies.

Although several candidates have been investigated in preclinical and early clinical phases for ARDS treatment, specific drugs have not been approved [2,43]. The major problem of drawing reliable conclusions from preclinical studies is that they often apply different LPS serotypes, doses, and routes of administration, and they lack positive controls.

The majority of studies do not report on respiratory function in the LPS-induced ALI model. The available data on lung function are quite contradictory, reporting on different parameters and changes [26,29]. The comparison and interpretation of respiratory parameters are also challenging due to the different measurement apparatuses using either unrestrained, restrained, or invasive techniques, spontaneously breathing or ventilated animals, as well as various study endpoints that can substantially influence the outcomes [27,29,44]. As such, a study using C57BL/6J mice and high dose (5 mg/kg) LPS reported similar decrease in tidal volume and minute ventilation, but opposite changes in frequency and peak expiratory flows, that might be attributed to the use of unrestrained whole-body plethysmograph as opposed to the restraint chambers used in our set of experiments [33].

Thus, the translational relevance of airway function might be debatable, due to the differences in the anatomy and physiological functions in mice and the various measurement techniques, changes in certain parameters do not necessarily reflect human pathophysiology [10].

Respiratory functional alterations are not exclusively and directly influenced by the cellular inflammatory mechanisms, but more by the peribronchial/perivascular edema formation. Minute ventilation, tidal volume, and peak expiratory flow rate were all reduced by dexamethasone, supported by its anti-edema action [45]. MPO, as an important but not exclusive enzyme involved in the complex inflammatory cascade induced by LPS, is produced by activated neutrophils and macrophages. Dexamethasone did not influence LPS-induced MPO activity, in agreement with the lack of effect on the accumulation of these inflammatory cells based on the histopathological results. Both macrophage- and neutrophil-released cytokines/chemokines and other mediators induce vasodilation and massive inflammatory cell recruitment, as well as the activation of peptidergic sensory nerves releasing substance P, calcitonin gene-related peptide (CGRP), pituitary adenylate cyclase-activating polypeptide (PACAP), etc. [41]. These complex inflammatory mechanisms with sensory-vascular-immune interactions lead to severe interstitial pneumonitis, which does not depend on the applied LPS dose, developing to a similar extent even in response to 0.25 mg/kg LPS, which is below the lowest (0.5 mg/kg) dose used in the literature.

However, our results highlight that LPS acutely induces distinct reproducible changes in mice, which are not strain-specific. Although baseline functional differences can be observed between the inbred C57BL/6J with a more homogenous genetic background and the outbred NMRI mice, these are most likely due to the difference in their initial body weight (20 ± 3 g and 30 ± 2 g, respectively) [46]. Larger NMRI females have greater tidal volume than control C57BL/6J mice. Greater tidal volume is usually coupled with lower breathing frequency, and minute ventilation is directly dependent on these parameters. However, the lung-to-body-weight ratio does not show a proportional relationship [47]. This is supported by the lung index (lung weight (g)/body weight (10 g)) of PBS-treated control animals, where we also observed strain differences. The lung index of control NMRI mice was lower than that of C57Bl/6J, which can be attributed to the significantly higher body mass index of the NMRI strain [48]. Despite these phenotypic differences, the direction and extent of LPS-induced alterations are quite consistent, as well as their response to a reference compound, supporting the translatability of these results to human lung conditions. Therefore, both strains are appropriate and applicable, validating the model for preclinical testing.

We also demonstrate valuable data regarding dose- and time-dependent LPS-induced thermoregulation changes. The initial hypothermia is mainly attributed to general (ketamine-xylazine) anesthesia, which peaks at 1–2 h after administration and recovers after 4–5 h in PBS-treated non-inflamed control mice. This effect is well-known, since both ketamine [49] and xylazine [50] were shown to decrease deep body temperature in rodents. The prominent anesthesia-induced transient drop in T_ab_ masks LPS-induced changes within the first hours; however, the dose-dependent changes are clearly detectable afterward.

Although the study endpoints are different, a drop in body temperature is described by several LPS ALI studies. Similar to our results in ketamine-xylazine-anaesthetized females, a 5 ± 2 °C decrease was observed in male mice at the 4-h timepoint [34]. In other studies, hypothermia in male but not female mice developed 1-4 h after 50 μg LPS under isoflurane anesthesia [22].

In our study, we chose the 24 h-timepoint based on the maximum of the inflammatory response and related functional alterations, which decrease later by the 48 h- and 72 h-timepoints. Tao and co-workers also demonstrated that lung injury already develops at 6 h, and becomes more severe by 12 h; however, its peak is observed at 24 h and is alleviated by 48 h and 72 h [21]. Another study also showed a similar pattern in body weight loss peaking at 24 h, then animals regaining weight gradually 4 days afterward [39]. Tsikis and co-workers found the highest tumor necrosis factor-α (TNF-α) and interleukin (IL)-6 levels in the bronchoalveolar lavage fluid (BALF) as well as pulmonary inflammatory infiltration at 24 h, which were decreased to control levels after 4 days, 7 days, and 4 weeks. However, they reported a progressive decrease in alveolarization, suggesting a long-term effect on structural alterations [26].

Our study has some limitations, namely the use of only female mice in contrast to most LPS studies using males [17,18]. However, based on our earlier experience using both sexes, the LPS-induced ALI model similarly develops in both sexes [51].

Others also revealed no sex difference in 3 mg/kg LPS-induced weight loss, BALF cell count, protein concentration, and lung lymphocytes [25]. Furthermore, a recent meta-analysis on sex-related differences in the mouse LPS-induced ALI model reported no difference in several inflammatory parameters, such as total protein concentration, IL-6, TNF-α, IL-1β concentrations, macrophage, and neutrophil counts in the BALF [52]. Few studies observed more severe lung histopathological inflammatory changes, BALF composition, and airway hyperresponsiveness in male mice, explained by the pro-inflammatory effects of testosterone, but they studied much earlier timepoints (6 h) [18,22].

Another caveat is that, based on our results, this model might not be optimal to differentiate on inflammation severity. Although the LPS model is not considered to be a disease model, its mechanism is well-established that TLR4 activation on macrophages induces a range of inflammatory mediator release leading to neutrophil accumulation, stimulation, and consequent cytokine storm characteristic of ARDS [53]. TLR4-dependent innate immune system activation is, therefore, a crucial step in regulating the response to bacterial infections. A wide range of adaptors and signal pathways have been described to interact with LPS-inducible TLR4 signaling [54]. However, TLR4-independent inflammasome activation by CD14 endocytosis has also been observed, which further contributes to innate immune recognition. It could potentially lead to overwhelming downstream activation [55]. Immunological dysregulation, therefore, seems to be the most plausible mechanism of glucocorticoid resistance in higher LPS doses. In vitro studies have shown certain pro-inflammatory cytokines, such as TNF-α, IL-1, and IFN-γ, to be associated with sustained glucocorticoid-resistance via shifting the expression of the glucocorticoid receptor toward the negative ϐ isoform [56,57]. These pro-inflammatory cytokines are known to be involved in the LPS-induced pneumonitis [44].

## 5. Conclusions

In summary, this study demonstrates that even low LPS doses induce characteristic functional and morphological inflammatory alterations in the lung, which do not worsen in response to even 20 times higher doses. Since the effect of pharmacological interventions is likely to be detectable in the case of the 0.25 mg/kg LPS dose, we suggest this protocol for testing novel anti-inflammatory agents.

## Figures and Tables

**Table 1:** Different LPS ALI model paradigms in the literature. (n.r.: not reported; n.u.: not used; i.p.: intraperitoneal; M: male; F: female; DEXA: dexamethasone).

LPS Dose	LPS Route of Admin	LPS Serotype	Strain	Sex	Endpoint	Anti-Inflammatory Drug Testing	Reference Compound	Reference
0.5 mg/kg (10 µg)	intratracheal	n.r.	C57BL/6J	M	24 h	Panaxydol	n.u.	Li et al., 2021 [13]
		n.r.	BALB/c	M	24 h	n.u.	n.u.	Wang et al., 2021 [14]
	intranasal	n.r.	BALB/c	M	day 7	Fuzhengjiedu formula	DEXA (1.5 mg/kg)	Lu et al., 2024 [15]
1 mg/kg	intratracheal	O111:B4	C57BL/6J, STAT6^flox/flox^	n.r.	24 h	n.u.	n.u.	Yang et al., 2022 [16]
		O111:B4	MMP3^+/+^, MMP3^−/−^	M, F	18 h	n.u.	n.u.	Puntorieri et al., 2016 [17]
		O111:B4	n.r.	M, F	6 h	n.u.	n.u.	Speyer et al., 2005 [18]
		n.r.	C57BL/6J	n.r.	16 h	n.u.	n.u.	Kovacs-Kasa et al., 2021 [19]
	intranasal	O55:B5	BALB/c	M	24 h	B7H3	n.u.	Li et al., 2016 [20]
2 mg/kg	intratracheal	O55:B5	C57BL/6J	M	6, 12, 24, 48, 72 h	erlotinib	n.u.	Tao et al., 2019 [21]
2.5 mg/kg (50 µg)	aspiration	O111:B4	C57BL/6J	M, F	6 h	n.u.	n.u.	Card et al., 2006 [22]
	inhalation	O55:B5	ICR	M	day 14	Xuanfei Baidu formula	DEXA (0.4 mg/kg)	Li Z et al., 2023 [23]
3 mg/kg	intratracheal	O111:B4	C57BL/6J, Trem2^−/−^	M	48 h	rhein, NFATc1 inhibitor	n.u.	Li X et al., 2023 [24]
		O55:B5	C57BL/6J	M, F	day 3, 7	n.u.	n.u.	Mock et al., 2023 [25]
75 µg	intratracheal	O111:B4	C57BL/6J	M	24 h, day 4, 7, 28	n.u.	n.u.	Tsikis et al., 2022 [26]
4 mg/kg	n.r.	n.r.	C57BL/6J	M	day 7	Xuanfei Baidu Decoction	DEXA (5 mg/kg)	Wang et al., 2022 [27]
5 mg/kg	intratracheal	O111:B4	C57BL/6J	M	12, 24 h	FGF1	n.u.	Dhlamini et al., 2022 [28]
		O111:B4	C57BL/6J	M	12 h	PTUPB	n.u.	Yang et al., 2020 [29]
		O111:B4	C57BL/6J	n.r.	48 h	fluorofenidone	n.u.	Lv et al., 2021 [30]
		O55:B5	CC10-rtTA/(tetO)7-Cre/cGAS^flox/flox^	n.r.	24 h	n.u.	n.u.	Jin et al., 2023 [31]
		O55:B5	ICR	M	72 h	Forsythiaside A	DEXA (5 mg/kg)	Wang et al., 2024 [32]
		n.r.	C57BL/6J	n.r.	24 h	valsartan	n.u.	Zhou et al., 2024 [33]
	intranasal	O111:B4	C57BL/6J, GP6^−/−^	M	4 h	JAQ1	n.u.	Burkard et al., 2023 [34]
		n.r.	C57BL/6J	M	24 h	PEP-sNASP peptide	n.u.	Wu et al., 2022 [35]
	i.p. injection	O111:B4	C57BL/6J	M	48 h	n.u.	n.u.	Chen et al., 2023 [36]
		n.r.	C57BL/6J	M	24 h	orexin-A	n.u.	Nie et al., 2023 [37]
	n.r.	n.r.	C57BL/6J, MD2^−/−^, TLR4^−/−^	M	6, 24 h	licochalcone A	n.u.	Zhu et al., 2023 [38]
180 µg; 3 × 60 µg	intranasal	n.r.	Bcl-2-(1/2)	M, F	day 1, 2, 3, 4	n.u.	n.u.	Tesfaigzi et al., 2001 [39]
15 mg/kg	Caudal vein	O55:B5	C57BL/6J, Nrf2^−/−^	M	12 h	n.u.	n.u.	Ma et al., 2023 [40]

## Data Availability

All data generated or analyzed during this study are included in this published article and its Supplementary Information Files. Further inquiries can be directed to the corresponding author.

## Supplementary Materials

The following supporting information can be downloaded at: https://www.mdpi.com/article/10.3390/biomedicines13061498/s1, Figure S1. LPS (0.25 mg/kg)-induced alterations in body weight, respiratory functions (frequency, tidal volume, minute ventilation, peak expiratory flow rate) lung index and interstitial inflammatory cell count in NMRI mice 24, 48 and 72 h after induction. n = 4–8/group (Effect size analysis, * Hedges’g > 0.8 vs PBS-treated group). Table S1. LPS (0.25 mg/kg)-induced alterations in body weight change, respiratory functions (frequency, tidal volume, minute ventilation, peak expiratory flow rate), lung index and inflammatory cell count in NMRI mice 24, 48, 72 hours after induction. g > 0.8 indicating large effect size calculated by Hedges’ g was considered significant. *p* < 0.05 analyzed by one-way ANOVA followed by Dunnett’s multiple comparisons test (respiratory function parameters, lung index, and inflammatory cell count), as well as repeated measures two-way ANOVA followed by Sidak’s post hoc test (body weight change) was considered statistically significant and marked as bold. n = 6–18 mice/group, * Hedges’g > 0.8 vs corresponding group. Table S2. LPS-induced alterations in respiratory functions (frequency, tidal volume, minute ventilation, peak expiratory flow rate) measured by non-invasive restraint plethysmogaph in response to dexamethasone. g > 0.8 indicating large effect size calculated by Hedges’ g was considered significant and *p* < 0.05 analyzed by one-way ANOVA followed by Dunnett’s multiple comparisons test (lung weight, respiratory function parameters, and MPO activity), two-way ANOVA followed by Tukey’s post hoc test (body weight change), or Kruskal-Wallis followed by Dunn’s post hoc test (histopathological semiquantitative scores) was considered statistically significant and marked as bold. n = 6–18 mice/group, * Hedges’g > 0.8 vs PBS; # Hedges’g > 0.8 corresponding LPS group. Table S3. Abdominal temperature changes over the course of 24 hours following 0.25, 1, 2.5, 5 mg/kg LPS treatment. Data represents *p* values analyzed by two-way ANOVA followed by Tukey’s post hoc test. n = 6–7/group. Table S4. LPS (0.25 mg/kg) -induced alterations in body weight change, total lung weight, composite score and MPO activity in response to dexamethasone in NMRI mice. g > 0.8 indicating large effect size calculated by Hedges’ g was considered significant and *p* < 0.05 analyzed by one-way ANOVA followed by Dunnett’s multiple comparisons test (lung weight, respiratory function parameters, and MPO activity), two-way ANOVA followed by Tukey’s post hoc test (body weight change), or Kruskal-Wallis followed by Dunn’s post hoc test (composite score) was considered statistically significant and marked as bold. n = 6–18 mice/group, * Hedges’g > 0.8 vs PBS; # Hedges’g > 0.8 corresponding LPS group. n = 5–6/group (Effect size analysis, * Hedges’g > 0.8 vs PBS; # Hedges’g > 0.8 vs corresponding LPS group). Table S5. Number of animals/group used in the series of experiments. Table S6. Number of animals/group included in the data analysis of studied parameters.

## Abbreviations

ALI	acute lung injury
ARDS	acute respiratory distress syndrome
BALF	bronchoalveolar lavage fluid
COVID-19	Coronavirus disease 2019
DEXA	dexamethasone
HE	hematoxylin-eosin
IL	interleukin
i.p.	intraperitoneal
LPS	lipopolysaccharide
MPO	myeloperoxidase
NAM	Non-Invasive Airway Mechanics
n.r.	not reported
n.u	not used
PBS	phosphate-buffered saline
SARS-CoV-2	Severe Acute Respiratory Syndrome Coronavirus-2
SEM	standard error of the mean
T_ab_	abdominal temperature
TLR4	toll-like receptor 4
TNF-α	tumor necrosis factor-α
Veh	Vehicle
