# Correction: Ritter et al. Optimization, Characterization and Pharmacological Validation of the Endotoxin-Induced Acute Pneumonitis Mouse Model. *Biomedicines* 2025, *13*, 1498

**DOI:** 10.3390/biomedicines13102384

**Published:** 2025-09-28

**Authors:** Emese Ritter, Kitti Hohl, László Kereskai, Ágnes Kemény, Dóra Hargitai, Veronika Szombati, Anikó Perkecz, Eszter Pakai, Andras Garami, Ákos Zsembery, Zsuzsanna Helyes, Kata Csekő

**Affiliations:** 1 Department of Pharmacology and Pharmacotherapy, Medical School, University of Pécs, Szigeti út 12, H-7624 Pécs, Hungary; rittermesi@gmail.com (E.R.); kitti.hohl.2000@gmail.com (K.H.); kemenyagnes1@gmail.com (Á.K.); veronika.szombati@aok.pte.hu (V.S.); perkecz@gmail.com (A.P.); helyes.zsuzsanna@pte.hu (Z.H.); 2 Department of Pathology, Medical School, University of Pécs, Szigeti út 12, H-7624 Pécs, Hungary; kereskai.laszlo@pte.hu; 3 Department of Pathology, Forensic and Insurance Medicine, Faculty of Medicine, Semmelweis University, Üllői út 93, H-1091 Budapest, Hungary; hargitai.dora@semmelweis.hu; 4 Department of Thermophysiology, Institute for Translational Medicine, Medical School, University of Pécs, Szigeti út 12, H-7624 Pécs, Hungary; eszter.pakai@aok.pte.hu (E.P.); andras.garami@aok.pte.hu (A.G.); 5 Department of Oral Biology, Faculty of Dentistry, Semmelweis University, Nagyvárad tér 4, H-1089 Budapest, Hungary; zsembery.akos@semmelweis.hu; 6 National Laboratory for Drug Research and Development, Magyar Tudósok krt. 2, H-1117 Budapest, Hungary; 7 HUN-REN-PTE Chronic Pain Research Group, H-7624 Pécs, Hungary; 8 PharmInVivo Hungary Ltd., Szondy György u 10, H-7629 Pécs, Hungary

## Error in Table

In the original publication [1], there was a mistake in Table 1 as published. As Ref. 14 has been retracted, it was deleted from the paper along with the related main text. Therefore, the third row of Table 1 was deleted. The corrected Table 1 appears below. The authors state that the scientific conclusions are unaffected. This correction was approved by the Academic Editor. The original publication has also been updated.

## Reference

As Ref. 14 has been retracted, it was deleted from the original paper [1]. With this correction, the order of some references has been adjusted accordingly. 

## Figures and Tables

**Table 1:** Different LPS ALI model paradigms in the literature. (n.r.: not reported; n.u.: not used; i.p.: intraperitoneal; M: male; F: female; DEXA: dexamethasone).

LPS Dose	LPS Route of Admin	LPS Serotype	Strain	Sex	Endpoint	Anti-Inflammatory Drug Testing	Reference Compound	Reference
0.5 mg/kg (10 µg)	intratracheal	n.r.	C57BL/6J	M	24 h	Panaxydol	n.u.	Li et al., 2021 [13]
	intranasal	n.r.	BALB/c	M	day 7	Fuzhengjiedu formula	DEXA (1.5 mg/kg)	Lu et al., 2024 [14]
1 mg/kg	intratracheal	O111:B4	C57BL/6J, STAT6^flox/flox^	n.r.	24 h	n.u.	n.u.	Yang et al., 2022 [15]
		O111:B4	MMP3^+/+^, MMP3^−/−^	M, F	18 h	n.u.	n.u.	Puntorieri et al., 2016 [16]
		O111:B4	n.r.	M, F	6 h	n.u.	n.u.	Speyer et al., 2005 [17]
		n.r.	C57BL/6J	n.r.	16 h	n.u.	n.u.	Kovacs-Kasa et al., 2021 [18]
	intranasal	O55:B5	BALB/c	M	24 h	B7H3	n.u.	Li et al., 2016 [19]
2 mg/kg	intratracheal	O55:B5	C57BL/6J	M	6, 12, 24, 48, 72 h	erlotinib	n.u.	Tao et al., 2019 [20]
2.5 mg/kg (50 µg)	aspiration	O111:B4	C57BL/6J	M, F	6 h	n.u.	n.u.	Card et al., 2006 [21]
	inhalation	O55:B5	ICR	M	day 14	Xuanfei Baidu formula	DEXA (0.4 mg/kg)	Li Z et al., 2023 [22]
3 mg/kg	intratracheal	O111:B4	C57BL/6J, Trem2^−/−^	M	48 h	rhein, NFATc1 inhibitor	n.u.	Li X et al., 2023 [23]
		O55:B5	C57BL/6J	M, F	day 3, 7	n.u.	n.u.	Mock et al., 2023 [24]
75 µg	intratracheal	O111:B4	C57BL/6J	M	24 h, day 4, 7, 28	n.u.	n.u.	Tsikis et al., 2022 [25]
4 mg/kg	n.r.	n.r.	C57BL/6J	M	day 7	Xuanfei Baidu Decoction	DEXA (5 mg/kg)	Wang et al., 2022 [26]
5 mg/kg	intratracheal	O111:B4	C57BL/6J	M	12, 24 h	FGF1	n.u.	Dhlamini et al., 2022 [27]
		O111:B4	C57BL/6J	M	12 h	PTUPB	n.u.	Yang et al., 2020 [28]
		O111:B4	C57BL/6J	n.r.	48 h	fluorofenidone	n.u.	Lv et al., 2021 [29]
		O55:B5	CC10-rtTA/(tetO)7-Cre/cGAS^flox/flox^	n.r.	24 h	n.u.	n.u.	Jin et al., 2023 [30]
		O55:B5	ICR	M	72 h	Forsythiaside A	DEXA (5 mg/kg)	Wang et al., 2024 [31]
		n.r.	C57BL/6J	n.r.	24 h	valsartan	n.u.	Zhou et al., 2024 [32]
	intranasal	O111:B4	C57BL/6J, GP6^−/−^	M	4 h	JAQ1	n.u.	Burkard et al., 2023 [33]
		n.r.	C57BL/6J	M	24 h	PEP-sNASP peptide	n.u.	Wu et al., 2022 [34]
	i.p. injection	O111:B4	C57BL/6J	M	48 h	n.u.	n.u.	Chen et al., 2023 [35]
		n.r.	C57BL/6J	M	24 h	orexin-A	n.u.	Nie et al., 2023 [36]
	n.r.	n.r.	C57BL/6J, MD2^−/−^, TLR4^−/−^	M	6, 24 h	licochalcone A	n.u.	Zhu et al., 2023 [37]
180 µg; 3 × 60 µg	intranasal	n.r.	Bcl-2-(1/2)	M, F	day 1, 2, 3, 4	n.u.	n.u.	Tesfaigzi et al., 2001 [38]
15 mg/kg	Caudal vein	O55:B5	C57BL/6J, Nrf2^−/−^	M	12 h	n.u.	n.u.	Ma et al., 2023 [39]
