# Supplementary material for: Optimization, Characterization and Pharmacological Validation of the Endotoxin-Induced Acute Pneumonitis Mouse Model
Source: Biomedicines. 2025 Jun 18;13(6):1498. doi: 10.3390/biomedicines13061498 (PMC12191197; doi:10.3390/biomedicines13061498)
Supplement: Supplementary file 1 [file biomedicines-13-01498-s001.zip › biomedicines-3597877-supplementary.pdf]

Supplementary material

Optimization, characterization and pharmacological validation of the endotoxin-induced acute pneumonitis mouse model

Emese Ritter, Kitti Hohl, László Kereskai, Ágnes Kemény, Dóra Hargitai, Veronika Szombati, Anikó Perkecz, Eszter Pakai, Andras Garami, Ákos Zsembery, Zsuzsanna Helyes†, Kata Csekő†

†These authors contributed equally to this work.

Corresponding author: Kata Csekő; email: cseko.kata@pte.hu; Tel: +36-72-538-212 (ext. 38230)

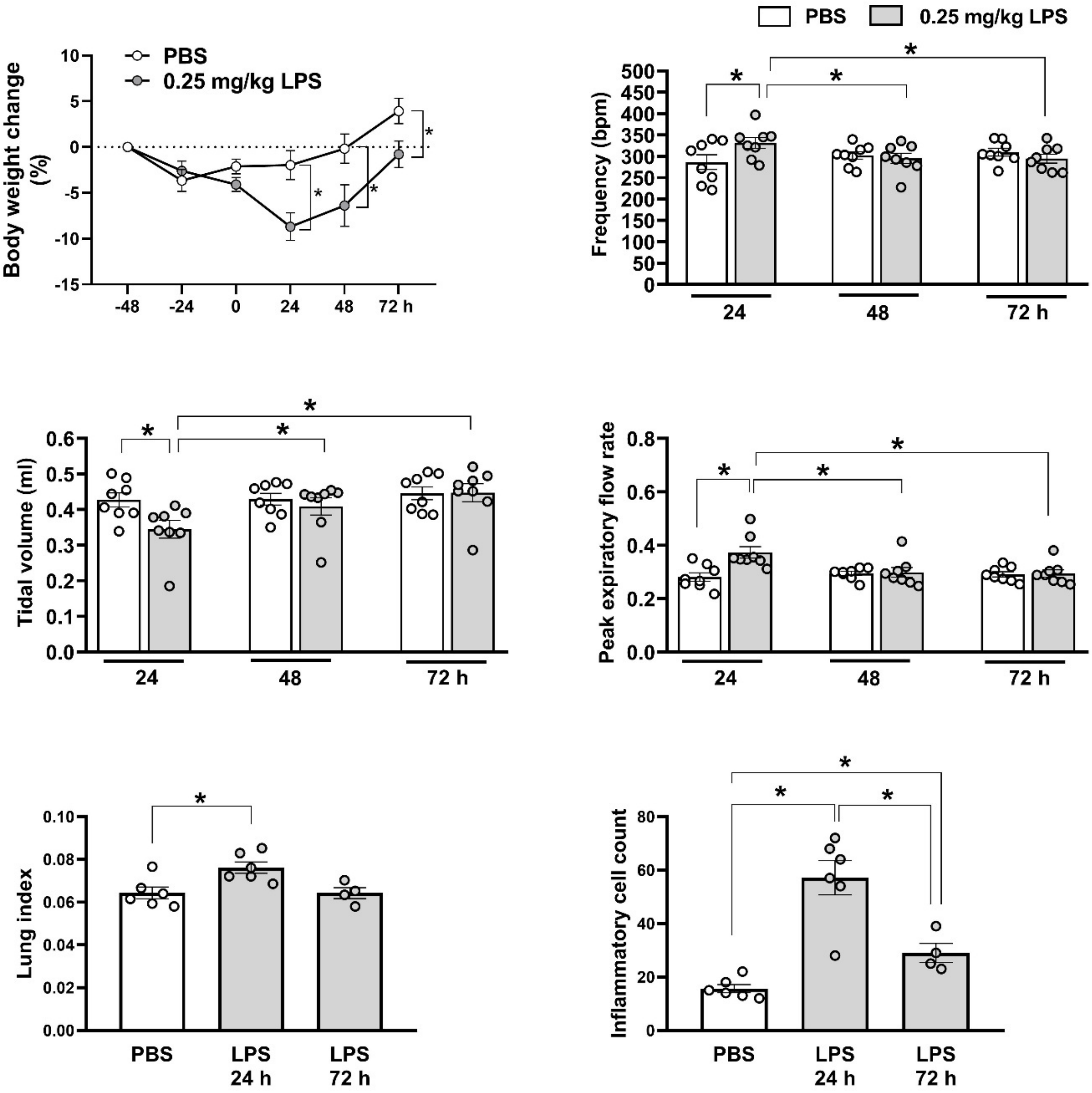

Figure S1. LPS (0.25 mg/kg)-induced alterations in body weight, respiratory functions (frequency, tidal volume, minute ventilation, peak expiratory flow rate) lung index and interstitial inflammatory cell count in NMRI mice 24, 48 and 72 h after induction. n=4-8/group (Effect size analysis, *Hedges'g > 0.8 vs PBS-treated group).

Table S1. LPS (0.25 mg/kg)-induced alterations in body weight change, respiratory functions (frequency, tidal volume, minute ventilation, peak expiratory flow rate), lung index and inflammatory cell count in NMRI mice 24, 48, 72 hours after induction. g>0.8 indicating large effect size calculated by Hedges' g was considered significant. p<0.05 analyzed by one-way ANOVA followed by Dunnett's multiple comparisons test (respiratory function parameters, lung index, and inflammatory cell count), as well as repeated measures two-way ANOVA followed by Sidak's post hoc test (body weight change) was considered statistically significant and marked as bold. n=6-18 mice/group, *Hedges'g > 0.8 vs corresponding group.

Body weight change				LPS (respective time-point)	PBS		LPS	LPS	
					48 h	72 h		48 h	72 h
	PBS	24 h	g	**1.45***	0.38	**1.33***	LPS	0.48	**1.79***
			p	**0.0368**	0.9056	0.550		0.6120	**0.0079**
		48 h	g	**1.06***	-	**0.92***		-	**0.98***
			p	0.1666		0.1395			0.1750
		72 h	g	**1.11***	-	-		-	-
			p	0.1319					

Frequency				LPS (respective time-point)	PBS		LPS	LPS	
					48 h	72 h		48 h	72 h
	PBS	24 h	g	**0.98***	0.38	0.56	LPS	**0.97***	**1.05***
			p	**0.0486**	0.8245	0.5264		0.1501	0.1354
		48 h	g	0.22	-	0.28		-	0.03
			p	0.9971		0.9950			>0.9999
		72 h	g	0.52	-	-		-	-
			p	0.8412					

Tidal volume				LPS (respective time-point)	PBS		LPS	LPS	
					48 h	72 h		48 h	72 h
	PBS	24 h	g	**1.23***	0.04	0.33	LPS	**0.87***	**1.37***
			p	**0.0438**	>0.9999	0.9609		**0.0368**	**0.0081**
		48 h	g	0.32	-	0.32		-	0.51
			p	0.9485		0.9767			0.6123
		72 h	g	0.02	-	-		-	-
			p	>0.9999					

Peak expiratory flow rate				LPS (respective time-point)	PBS		LPS	LPS	
					48 h	72 h		48 h	72 h
	PBS	24 h	g	**1.64***	0.34	0.25	LPS	**0.83***	**1.45***
			p	0.0005	0.9564	0.9887		**0.0055**	**0.0031**
		48 h	g	0.06	-	0.11		-	0.06
			p	0.9997		0.9997			0.9997
		72 h	g	0.09	-	-		-	-
			p	>0.9999					

Lung index			LPS	
			24 h	72 h
	PBS	g	**1.63***	0.65
		p	**0.0124**	0.9993
	LPS 24 h	g	-	0.53
		p		0.0224

Inflammatory cell count		LPS	
		24 h	72 h
	PBS	**3.34***	**2.29***
		**<0.0001**	0.1307
	LPS 24 h	-	1.92*
			0.0023

Table S2. LPS-induced alterations in respiratory functions (frequency, tidal volume, minute ventilation, peak expiratory flow rate) measured by non-invasive restraint plethysmogaph in response to dexamethasone. *g*>0.8 indicating large effect size calculated by Hedges' g was considered significant and p<0.05 analyzed by one-way ANOVA followed by Dunnett's multiple comparisons test (lung weight, respiratory function parameters, and MPO activity), two-way ANOVA followed by Tukey's post hoc test (body weight change) or Kruskal-Wallis followed by Dunn's post hoc test (histopathological semiquantitative scores) was considered statistically significant and marked as bold. n = 6-18 mice/group, *Hedges'g > 0.8 vs PBS; # Hedges'g > 0.8 corresponding LPS group.

	Body weight change		**LPS (mg/kg)**			
			**0.25**	**1**	**2.5**	**5**
**PBS**		g	**1.48***	**3.45***	**4.04***	**3.03***
		p	0.0537	**0.0013**	**0.0004**	**<0.0001**
**DEXA**		g	**0.88 #**	**1.36 #**	2.51 #	0.06
		p	0.3068	0.0565	0.0006	0.9900

	Lung index		**LPS (mg/kg)**			
			**0.25**	**1**	**2.5**	**5**
**PBS**		g	**2.10***	**2.71***	**5.61***	**3.68***
		p	**0.0021**	**<0.0001**	**<0.0001**	**<0.0001**
**DEXA**		g	**2.01 #**	**0.93 #**	**1.78 #**	0.69
		p	**0.0045**	0.1728	**0.0053**	0.2436

	Frequency		**LPS (mg/kg)**			
			**0.25**	**1**	**2.5**	**5**
**PBS**		g	**1.92***	**2.03***	**2.76***	**1.13***
		p	**0.0089**	**0.0163**	**0.0005**	**0.0295**
**DEXA**		g	0.4	0.55	**0.80 #**	0.46
		p	0.7440	0.4854	0.1525	0.5509

	Tidal volume		**LPS (mg/kg)**			
			**0.25**	**1**	**2.5**	**5**
**PBS**		g	**4.75***	**5.57***	**6.84***	**8.34***
		p	**<0.0001**	**<0.0001**	**<0.0001**	**<0.0001**
**DEXA**		g	**2.24 #**	**0.81 #**	**0.98 #**	0.71
		p	**0.0007**	0.2000	0.0855	0.3110

	Minute ventilation		**LPS (mg/kg)**			
			**0.25**	**1**	**2.5**	**5**
**PBS**		g	**1.77***	**1.97***	**4.06***	**2.53***
		p	**0.0230**	**0.0144**	**0.0003**	**<0.0001**
**DEXA**		g	**1.40 #**	**0.80 #**	0.63	0.15
		p	**0.0258**	0.1978	0.3022	0.9464

	Peak expiratory flow rate		**LPS (mg/kg)**			
			**0.25**	**1**	**2.5**	**5**
**PBS**		g	**3.32***	**3.71***	**3.27***	**1.40***
		p	**<0.0001**	**<0.0001**	**0.0020**	**0.0103**
**DEXA**		g	**1.62 #**	0.12	0.29	0.47
		p	**0.0375**	0.9706	0.7412	0.4749

	Perivascular oedema		**LPS (mg/kg)**			
			**0.25**	**1**	**2.5**	**5**
**PBS**		g	**5.96***	0.75	**2.20***	**1.19***
		p	**<0.0001**	0.4388	**0.0012**	**0.0244**
**DEXA**		g	**0.97 #**	0.21	0.51	**0.80 #**
		p	**0.0223**	0.6041	0.3505	0.2119

	Eosinophil macrophages		**LPS (mg/kg)**			
			**0.25**	**1**	**2.5**	**5**
**PBS**		g	**1.56***	**1.04***	0.54	0.27
		p	**0.0042**	**0.0350**	0.3828	0.9755
**DEXA**		g	**0.98 #**	**1.11#**	0.44	0.15
		p	0.3992	0.2927	0.8403	>0.9999

	Granulocytes		**LPS (mg/kg)**			
			**0.25**	**1**	**2.5**	**5**
**PBS**		g	**2.73***	**1.30***	**2.12***	**1.68***
		p	**0.0029**	**0.0296**	**0.0063**	**0.0061**
**DEXA**		g	0.14	0.13	0.25	0.41
		p	>0.9999	>0.9999	>0.9999	>0.9999

	Composite score		**LPS (mg/kg)**			
			**0.25**	**1**	**2.5**	**5**
**PBS**		g	**3.21***	**1.27***	**1.77***	**1.07***
		p	**0.0007**	**0.0226**	**0.0062**	0.0634
**DEXA**		g	**0.89 #**	0.40	0.04	0.33
		p	0.5726	>0.9999	>0.9999	>0.9999

	MPO activity		**LPS (mg/kg)**			
			**0.25**	**1**	**2.5**	**5**
**PBS**		g	**1.73***	**3.28***	**1.77***	**2.17***
		p	**0.0003**	**<0.0001**	**0.0004**	**<0.0001**
**DEXA**		g	0.28	0.58	0.41	0.64
		p	0.6741	0.2007	0.3915	0.1658

Table S3. Abdominal temperature changes over the course of 24 hours following 0.25, 1, 2.5, 5 mg/kg LPS treatment. Data represents *p* values analyzed by two-way ANOVA followed by Tukey's post hoc test. n=6-7/group

**Time (h)**	**LPS (mg/kg)**									
	**0.25**	**1**	**2.5**	**5**	**0.25**	**1**	**2.5**	**0.25**	**1**	**0.25**
	**vs PBS**				**vs 5**			**vs 2.5**		**vs 1**
**-5**	0.9999	0.9853	0.8762	0.7356	0.6813	0.9605	0.2502	0.9288	0.6376	0.9694
**-4**	0.942	>0.9999	0.9999	0.9317	>0.9999	0.9723	0.9113	0.9221	0.9994	0.9779
**-3**	0.707	0.9541	0.996	0.9987	0.8790	0.8757	>0.9999	0.9029	0.8292	0.3091
**-2**	0.9698	0.9928	0.9712	0.9962	0.8337	>0.9999	0.8461	>0.9999	0.7777	0.7551
**-1**	0.8522	0.878	0.7576	0.9753	0.1446	0.1501	0.1467	0.9899	0.9703	0.9997
**0**	0.2114	0.4446	0.7547	0.8368	0.8441	0.9851	>0.9999	0.8110	0.9860	0.9637
**1**	0.0702	0.9654	0.9998	0.9956	0.3775	0.9998	0.9986	0.0765	0.9803	0.3850
**2**	0.968	0.8283	0.8825	0.8268	0.9996	0.9986	>0.9999	0.9997	0.9997	0.9967
**3**	0.1779	0.4307	0.1571	0.4418	0.6515	0.9938	0.6322	0.9996	0.9277	0.8950
**4**	0.0502	**0.0047***	**0.0019***	**0.0012***	0.5013	0.5459	0.8669	0.8366	0.9445	0.9820
**5**	**0.0005***	**0.0004***	**0.0002***	**<0.0001***	0.6465	0.8354	0.3723	0.9940	0.9359	0.9965
**6**	**<0.0001***	**0.0002***	**0.0001***	**<0.0001***	0.8841	0.8911	0.5141	0.9400	0.9704	>0.9999
**7**	**0.0001***	**<0.0001***	**<0.0001***	**<0.0001***	0.8149	0.6960	0.7973	>0.9999	0.9999	>0.9999
**8**	**0.0010***	**0.0002***	**<0.0001***	**<0.0001***	0.8585	0.3185	0.9973	0.9155	0.2885	0.9826
**9**	**0.0144***	**0.0005***	**<0.0001***	**<0.0001***	0.8452	0.2085	0.9891	0.6434	**0.0409 #**	0.9709
**10**	**0.0365***	**0.0020***	**<0.0001***	**0.0008***	0.7949	0.0978	>0.9999	0.7421	**0.0320 #**	0.8932
**11**	0.0865	**0.0063***	**0.0002***	**0.0010***	0.3994	**0.0109 #**	>0.9999	0.3586	**0.0043 #**	0.7442
**12**	0.1645	**0.0097***	**0.0020***	**0.0036***	0.3360	**0.0189 #**	0.9998	0.2886	**0.0137 #**	0.8385
**13**	0.2694	**0.0110***	**0.0119***	**0.0151***	0.5140	0.0627	0.9947	0.3800	**0.0492 #**	0.8426
**14**	0.4780	**0.0066***	**0.0299***	**0.0212***	0.5002	0.0784	0.9997	0.4838	0.1053	0.9530
**15**	0.3126	0.0548	**0.0477***	**0.0288***	0.0917	0.0777	0.9639	0.2241	0.1681	0.9996
**16**	0.1750	**0.0051***	0.0644	**0.0213***	0.0745	0.0738	0.8210	0.3541	0.3068	>0.9999
**17**	0.2620	**0.0042***	**0.0350***	**0.0315***	0.0959	0.1412	0.9346	0.1847	0.2718	0.9137
**18**	**0.0193***	**<0.0001***	**0.0116***	**0.0214***	0.0861	0.1652	0.9379	0.1005	0.2438	0.4587
**19**	**0.0378***	**<0.0001***	**0.0216***	**0.0368***	0.0925	0.2953	0.8882	0.1005	0.5561	**0.0214 #**
**20**	0.3780	**0.0049***	**0.0119***	0.1099	0.1864	0.4621	0.9708	**0.0367 #**	0.3126	0.0926
**21**	0.1429	**0.0008***	**0.0093***	0.1041	0.2409	0.5304	0.9495	0.0697	0.4723	0.1433
**22**	**0.0444***	**0.0003***	**0.0052***	0.0710	0.2083	0.5036	0.9464	0.0556	0.4521	0.1369
**23**	0.2038	**0.0030***	**0.0369***	0.1298	0.3327	0.6065	0.9280	0.2927	0.8058	0.3431
**24**	0.3060	**0.0007***	**0.0197***	0.1844	0.3805	0.7465	0.9259	0.1920	0.9087	0.1989
**25**	0.2946	**0.0027***	**0.0432***	0.1286	0.2289	0.5610	0.6629	0.2347	0.9982	0.0609

Table S4. LPS (0.25 mg/kg) -induced alterations in body weight change, total lung weight, composite score and MPO activity in response to dexamethasone in NMRI mice. g>0.8 indicating large effect size calculated by Hedges' g was considered significant and p<0.05 analyzed by one-way ANOVA followed by Dunnett's multiple comparisons test (lung weight, respiratory function parameters, and MPO activity), two-way ANOVA followed by Tukey's post hoc test (body weight change), or Kruskal-Wallis followed by Dunn's post hoc test (composite score) was considered statistically significant and marked as bold. n = 6-18 mice/group, *Hedges'g > 0.8 vs PBS; # Hedges'g > 0.8 corresponding LPS group.n=5-6/group (Effect size analysis, *Hedges'g > 0.8 vs PBS; # Hedges'g > 0.8 vs corresponding LPS group).

	Parameter		LPS (mg/kg)	Parameter		LPS (mg/kg)
	**Body weight change**		**0.25**	**Lung index**		**0.25**
**PBS**		g	**1.43***		g	**7.87***
		p	0.0536		p	**<0.0001**
**DEXA**		g	0.53		g	**2.47 #**
		p	0.5342		p	**0.0002**
	**Minute ventilation**		**0.25**	**Peak expiratory flow rate**		**0.25**
**PBS**		g	**1.23***		g	**1.13***
		p	**0.1992**		p	0.0894
**DEXA**		g	**3.40 #**		g	**1.28 #**
		p	**0.0014**		p	0.1534
	**Frequency**		**0.25**	**Tidal volume**		**0.25**
**PBS**		g	**1.95***		g	**2.02***
		p	**0.0098**		p	**0.0080**
**DEXA**		g	0.36		g	**1.61 #**
		p	0.7329		p	**0.0391**
	**Composite score**		**0.25**	**MPO activity**		**0.25**
**PBS**		g	**1.03***		g	**1.39***
		p	**0.0133**		p	**0.0419**
**DEXA**		g	**0.97 #**		g	**1.05 #**
		p	0.1102		p	0.0893

Table S5. Number of animals/group used in the series of experiments.

<table>
<tr><th>Dose-response study in C57BL/6J</th><th>PBS</th><th>LPS</th><th>LPS + DEXA</th><th colspan="3">outcome measures</th></tr>
<tr><td>0.25 g/kg LPS</td><td>5</td><td>6</td><td>7</td><td colspan="3" rowspan="4">- body weight change
- lung index
- respiratory functions
- histopathology
- MPO activity</td></tr>
<tr><td>1 mg/kg LPS</td><td>5</td><td>7</td><td>7</td></tr>
<tr><td>2.5 mg/kg LPS</td><td>4</td><td>8</td><td>8</td></tr>
<tr><td>5 mg/kg LPS</td><td>4</td><td>10</td><td>6</td></tr>
<tr><th>Abdominal temperature study in C57BL/6J</th><th>PBS</th><th>0.25 mg/kg LPS</th><th>1 mg/kg LPS</th><th>2.5 mg/kg LPS</th><th>5 mg/kg LPS</th><th>outcome measures</th></tr>
<tr><td></td><td>7</td><td>6</td><td>6</td><td>7</td><td>6</td><td>- abdominal temperature
- MPO activity</td></tr>
<tr><th>Strain-dependent alterations in NMRI</th><th>PBS</th><th>LPS</th><th>LPS + DEXA</th><th colspan="3">outcome measures</th></tr>
<tr><td>0.25 g/kg LPS</td><td>6</td><td>7</td><td>7</td><td colspan="3">- body weight change
- lung index
- respiratory functions
- histopathology
- MPO activity</td></tr>
<tr><th>Time-dependent alterations in NMRI</th><th>PBS</th><th>LPS 24 h</th><th>LPS 72 h</th><th colspan="3">outcome measures</th></tr>
<tr><td>0.25 g/kg LPS</td><td>8</td><td>8</td><td>8</td><td colspan="3">- body weight change
- lung index
- respiratory functions
- histopathology</td></tr>
</table>

Table S6. Number of animals/group included in the data analysis of studied parameters.

**C57BL/6J (Fig. 1-5)**	**PBS**	**0.25 mg/kg LPS**	**0.25 mg/kg LPS + DEXA**	**1 mg/kg LPS**	**1 mg/kg LPS + DEXA**	**2.5 mg/kg LPS**	**2.5 mg/kg LPS + DEXA**	**5 mg/kg LPS**	**5 mg/kg LPS + DEXA**
**body weight change**	18	6	7	7	7	8	8	10	6
**lung index**	18	6	7	7	7	8	8	10	6
**respiratory functions**	18	6	7	7	7	8	8	8	6
**abdominal temperature**	7	6	-	6	-	7	-	6	-
**histopathology**	18	6	7	7	7	8	8	8	6
**MPO activity**	20	11	7	12	7	13	8	12	6

**NMRI (Fig. 6-7)**	**PBS**	**0.25 mg/kg LPS**	**0.25 mg/kg LPS + DEXA**
**body weight change**	6	7	7
**lung index**	6	7	7
**histopathology**	6	7	7
**MPO activity**	5	6	6
**respiratory functions**	5	6	6

**NMRI (Suppl. Fig. 1)**	**PBS – 24 h**	**0.25 mg/kg LPS – 24 h**	**PBS – 48 h**	**0.25 mg/kg LPS – 48 h**	**PBS – 72 h**	**0.25 mg/kg LPS – 72 h**
**body weight change**	8	8	8	8	8	8
**respiratory functions**	8	8	8	8	8	8
**lung index**	6	6	-	-	-	4
**histopathology**	6	6	-	-	-	4
